# Transgene-design: a web application for the design of mammalian transgenes

**DOI:** 10.1093/bioinformatics/btac139

**Published:** 2022-03-04

**Authors:** Stefanie Mühlhausen, Laurence D Hurst

**Affiliations:** Milner Centre for Evolution, Department of Biology and Biochemistry, University of Bath, Bath BA2 7AY, UK; Milner Centre for Evolution, Department of Biology and Biochemistry, University of Bath, Bath BA2 7AY, UK

## Abstract

**Summary:**

Transgene-design is a web application to help design transgenes for use in mammalian studies. It is predicated on the recent discovery that human intronless transgenes and native retrogenes can be expressed very effectively if the GC content at exonic synonymous sites is high. In addition, as exonic splice enhancers resident in intron containing genes may have different utility in intronless genes, these can be reduced or increased in density. Input can be a native gene or a commercially ‘optimised’ gene. The option to leave in the first intron and to protect or avoid other motifs is also permitted.

**Availability and implementation:**

Transgene-design is based on a ruby for rails platform. The application is available at https://transgene-design.bath.ac.uk. The code is available under GNU General Public License from GitHub (https://github.com/smuehlh/transgenes).

**Supplementary information:**

Supplementary data are available at *Bioinformatics* online.

## 1 Introduction 

The average human gene is over 90% intron (Piovesan *et al.*, 2019), more than in most other species (Warnecke *et al.*, 2008). As a consequence, to make manageable transgenes (e.g. by gene synthesis) for experimentation or for gene therapy it is necessary to remove introns. Typically, however, the long first intron is often retained as this is necessary for robust gene expression (Le Hir *et al.*, 2003). Indeed, intronless transgenes are classically avoided as they express poorly (see e.g. Brinster *et al.*, 1988). To both save synthesis costs and to increase the CDS lengths that are stable via synthesis, it would be advantageous to be able to robustly express intronless transgenes. 

We recently observed that human intronless retrogenes have much higher GC content at synonymous sites compared to their intron containing parental genes (Mordstein *et al.*, 2020). Stimulated by this, we showed, using synthetic transgenes differing in synonymous GC content, that poor expression of intronless transgenes can be very effectively mitigated by increasing such GC content (Mordstein *et al.*, 2020). The effect is particular to the 5′ end of the gene (Mordstein *et al.*, 2020). The mechanism by which high GC rescues intronless transgenes is not fully resolved but relates in part to nuclear export control (Mordstein *et al.*, 2020; Palazzo *et al.*, 2012, 2021; Zuckerman *et al.*, 2020), possibly as part of an evolved quality control system e.g. to filter out spurious non-coding transcripts (Palazzo *et al.*, 2012) or viral sequences (Mordstein *et al.*, 2021).

The removal of introns also potentially comes with its own issues. Possibly because of the size and number of introns, humans have unusually high densities of exonic splice enhancers (ESEs) towards the ends of their exons (Fairbrother *et al.*, 2004a,b; Warnecke *et al.*, 2008; Wu *et al.*, 2015). These hexameric motifs attract SR proteins and in so doing direct the SR protein to an exon–intron junction to initiate splicing (Blencowe, 2000). In intronless human retrogenes rates of evolution are highest in proximity to the paralogous locations of exon–intron junctions in the intron containing parental gene (Parmley *et al.*, 2007). This is potentially consistent with selection to reduce ESE–SR protein attraction as splicing is deleterious in intronless genes although there was no evidence that ESEs were especially being degraded (Parmley *et al.*, 2007). Indeed, native human intronless genes retain ESEs and these remain under purifying selection suggesting non-splicing roles (Savisaar *et al.*, 2016). Preliminary data (unpublished) finds that modification of ESE density alters expression level, independent of GC effects, but at a potential cost to increased missplicing of the intronless transcript. Manipulation of ESE density may well be helpful for both intronless and single intron transgenes.

In the above context, it is helpful, particularly for especially long CDS genes, to make effective intronless versions. We here describe a web application (https://transgene-design.bath.ac.uk) to (i) adjust GC content and (ii) to modify ESE density of intronless genes. It also permits the first intron to be retained if desired and important motifs to be protected or avoided.

## 2 Features

The application permits the user to upload a desired 5′ or 3′ sequence and an accompanying gene body. The gene can be input from a Genbank or fasta file (paste or upload). The user then has an option to enter ESE motifs via paste function, upload or use any one of five predefined sets. These are RESCUE-ESE (Fairbrother *et al.*, 2004a,b), ESR (Goren *et al.*, 2006), Ke-ESE400 (Ke *et al.*, 2011), PESE (Zhang *et al.*, 2004) or the set of hexameric ESEs found in at least three of the four, INT3 (Caceres *et al.*, 2013). For further characterization and comparison of these sets, see Caceres *et al.* (2013).

The user can select to increase or decrease ESE content (see ‘more options’). In the latter case, the application identifies ESEs to be removed and does so in line with GC stipulations. In our experience it is possible to reduce ESE density but never to remove them entirely, which is likely to be beneficial (Savisaar *et al.*, 2016). The user can define motifs to be left intact or to not introduce (e.g. known miRNA binding sites).

Finally, to adjust GC content the user can (i) opt to just adjust ESEs not GC (not recommended for most genes as GC3 is typically too low), (ii) match the GC at synonymous sites to that seen in 1 or 2 exon genes as a function of 5′ proximity, (iii) maximize GC or (iv) humanize (match human codon usage). For intronless genes we recommend either options 2 or 3. For genes retaining the first intron we recommend option 2.

To match GC content to that seen in 1 or 2 exon genes, synonymous codons are scored to position-dependent codon favourability in human genes. Codon favourability is derived from a matrix of probabilities for the last nucleotide in a codon box (6-fold codon boxes being split into their respective 4-fold and 2-fold sub-boxes), fitted into curves as a function of position. Alternatively, GC content may be adjusted to position-independent, human codon usage obtained from the Codon Usage Database (Nakamura *et al.*, 2000) (https://www.kazusa.or.jp/codon/) or by simply favouring codons ending in G or C. In any case, synonymous codons are scored against the respective GC target. For each site, one of the synonymous codons is selected with a likelihood corresponding to its score.

To adjust ESE content, synonymous codons in the vicinity of the discarded introns (with vicinity being defined as up to 70 nucleotides) are tested for their ESE resemblance. This adds to the codons’ likelihood for being selected for a given position. In case the user opted to adjust only ESE resemblance but not GC content, there is no GC-based component to this likelihood. Testing codons for their ESE resemblance may optionally be expanded to all codons, including those in exon cores. Per default, all but the first intron are discarded. Optionally, the user may specify to remove the first intron, too. Testing a synonymous codon for its ESE resemblance is compatible with any of the above mentioned options to adjust GC content.

Restriction sites are used to fine-tune which synonymous sites are inspected. Those sites that are part of a restriction enzyme the user wishes to retain are skipped while iterating through the sequence. Motifs that should be avoided are used as a blacklist against which a synonymous codon selected for its GC content/ESE resemblance is checked: if a pre-selected codon would introduce a motif on that list, the codon will not be used and the site will be left as is. For illustration of workflow, see Supplementary Figure S1.

The application generates a variant cloud in line with target GC content, ESE and restriction enzyme resemblance. It moves over the gene’s exonic sequence, inspecting one synonymous site at a time. A codon is retained or replaced by one of its synonymous alternatives based on the GC and/or ESE score it achieves. To ensure non-deterministic codon selection, codon scores are converted into a selection likelihood. At 6-fold degenerate sites all six synonymous codons are considered by default. The user can optionally adjust this behaviour such that only codons of the respective 2- or 4-codon sub-box are considered. Repeating this sliding window approach, the variant cloud gets populated. The variant best matching aforementioned targets is displayed on a results page. All other variants are available for download. Given that 50% of nucleotides in ESEs are A (25% G), highly GC rich sequences tend not to be commensurate with high ESE densities.

## Supplementary Material

btac139_Supplementary_Data
