# Supplementary material for: Transgene-design: a web application for the design of mammalian transgenes
Source: Bioinformatics. 2022 Mar 4;38(9):2626–7. doi: 10.1093/bioinformatics/btac139 (PMC9048660; doi:10.1093/bioinformatics/btac139)
Supplement: btac139_Supplementary_Data [file btac139_supplementary_data.pdf]

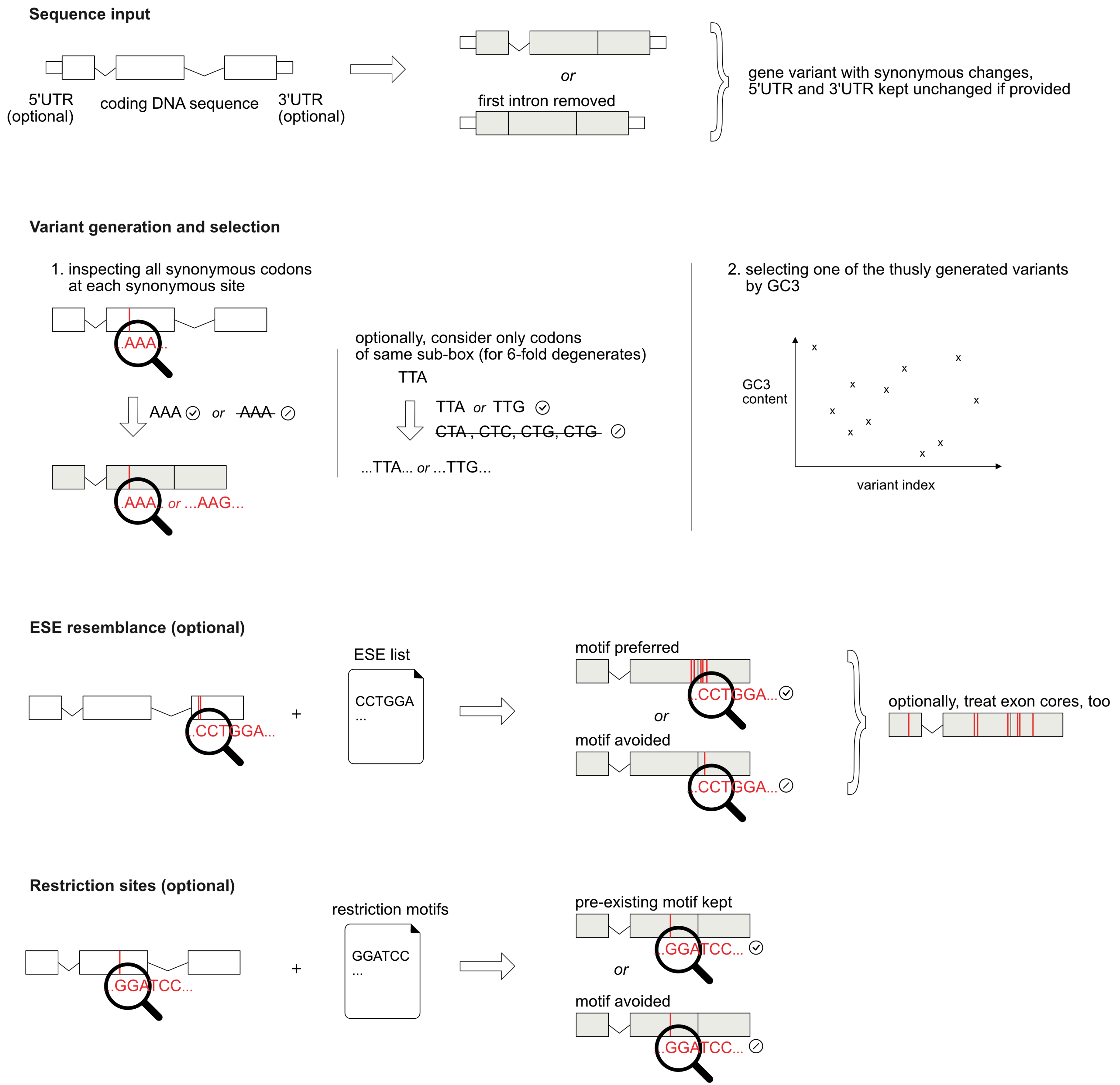

Sequence input
5'UTR (optional)
coding DNA sequence
3'UTR (optional)
or
first intron removed
gene variant with synonymous changes, 5'UTR and 3'UTR kept unchanged if provided
Variant generation and selection
1. inspecting all synonymous codons at each synonymous site
..AAA..
AAA or AAA
..AAA.. or ...AAG...
optionally, consider only codons of same sub-box (for 6-fold degenerates)
TTA
TTA or TTG
CTA, CTC, CTG, CTG
...TTA... or ...TTG...
2. selecting one of the thusly generated variants by GC3
GC3 content
variant index
ESE resemblance (optional)
..CCTGGA...
+
ESE list
CCTGGA
...
motif preferred
..CCTGGA...
or
motif avoided
..CCTGGA...
optionally, treat exon cores, too
Restriction sites (optional)
..GGATCC...
+
restriction motifs
GGATCC
...
pre-existing motif kept
..GGATCC...
or
motif avoided
..GGATCC...
